# Development and external validation of a prognostic nomogram for event-free survival in resectable non-small cell lung cancer after neoadjuvant chemoimmunotherapy

**DOI:** 10.3389/fonc.2025.1682497

**Published:** 2025-12-16

**Authors:** Linlin Qu, Wei Sun, Xinying Liu, Jiting Di, Suxia Wang, Yan Xiong

**Affiliations:** 1 Department of Pathology, Peking University First Hospital, Beijing, China; 2 Department of Pathology, Peking University People’s Hospital, Beijing, China; 3 Department of Pathology, Peking University Cancer Hospital & Institute, Key Laboratory of Carcinogenesis and Translational Research (Ministry of Education), Beijing, China; 4 Laboratory of Electron Microscopy, Peking University First Hospital, Beijing, China

**Keywords:** non-small cell lung cancer, postoperative prognostic factors, neoadjuvant chemoimmunotherapy, major pathologic response, nomogram model

## Abstract

**Background:**

Combining multiple prognostic factors can enhance risk assessment for resected non-small cell lung cancer (NSCLC). Our aim was to evaluate the prognostic significance of ypT staging, ypN staging, major pathologic response (MPR) and pathology response in lymph node, and to develop a combined prognostic model to predicting event-free survival in resectable NSCLC patients after neoadjuvant chemoimmunotherapy (NCI).

**Methods and results:**

Two independent cohorts (derivation, n=208; external validation, n=91) were utilized. Pathologic assessment and ypTNM staging followed recommendations from the International Association for the Study of Lung Cancer (IASLC) and the 8th edition AJCC TNM classification. The evaluation of lymph node is documented according to IASLC recommendations, and the mean metastatic tumor size (MTS) in lymph node was evaluated in each case. MPR was defined as ≤10% residual visible tumor in the tumor bed. The survival endpoint was event-free survival (EFS). Univariate and multivariate survival analyses identified nonMPR (HR, 2.860; 95% CI, 1.245-6.567, p=0.013), ypT3/4 (HR, 3.987; 95% CI, 1.496-10.629, p=0.006), and MTS (MTS ≤ 4.5mm vs negative; HR, 4.059; 95% CI, 1.558-10.571, p=0.004; MTS>4.5mm vs negative; HR, 6.871; 95% CI, 1.713-27.564, p=0.007) were independent adverse prognostic factors in the derivation cohort. We built a nomogram model including ypT stage, MPR and pathology response in lymph node to predict EFS, demonstrating high efficacy in both derivation (the Area Under Curve, AUC = 0.77) and external validation cohorts (AUC = 0.72). The Risk Stratification System showed that the EFS of low-risk patients was considerably better than that of high-risk patients (P < 0.0001).

**Conclusions:**

Prognostic model integrating ypT staging, MPR and the mean MTS improves EFS prediction for NSCLC following NCI.

## 1 Introduction

Neoadjuvant chemoimmunotherapy (NCI) has been developed in recent years, with numerous clinical trials demonstrating its safety and effectiveness in non-small cell lung cancer (NSCLC) (1–4). While previous studies have primarily focused on biomarkers like PD-L1 (2), tumor mutational burden (5), and tumor infiltrating lymphocytes (5) to assess tumor response, there is a continued need for reliable clinicopathological factors for postoperative risk stratification.

Pathologic response and the ypTNM system (pathological staging after neoadjuvant therapy) are regarded as crucial postoperative prognostic factors. The significance of ypTNM classification and the response of lymph node metastasis for EFS is still controversial. So far, major pathologic response (MPR), defined as ≤10% residual visible tumor in resected specimens (6, 7), is the only well recognized predictor for postoperative event-free survival (EFS) of patients treated with NCI (9, 10). Additionally, the authors suggested that lymph node pathologic response can offer supplementary prognostic insights beyond those from the primary tumor alone (9). However, a universally accepted method for lymph node metastasis (LNM) pathology evaluation remains absent. The metastatic tumor size (MTS) in lymph nodes is recorded based on the IASLC guideline (6), whereas the percentage of residual viable tumor (RVT%) is guided by immune-related pathologic response criteria (irPRC) (7, 8). Importantly, the optimal cutoffs for MTS and RVT% in predicting survival after NCI in NSCLC are yet to be established.

The aim of this study is to investigate the prognostic significance of ypTNM classification, MPR, and response of lymph node metastasis, to develop a nomogram model for better prediction of EFS in resectable NSCLC patients following NCI.

## 2 Materials and methods

### 2.1 Patients

This multicenter, retrospective study was conducted at Beijing Cancer Hospital and Peking University People’s Hospital in Beijing, China. Two independent cohorts were assembled: the derivation cohort, consisting of patients with NSCLC who received neoadjuvant immunotherapy combined with chemotherapy at Beijing Cancer Hospital (from January 2019 to August 2022), and the external validation cohort from Peking University People’s Hospital (from August 2019 to October 2022). All patients underwent complete surgical resection, including pneumonectomy, lobectomy, and wedge resection. Patients without complete follow-up and/or standardized treatment were excluded from the study (**Supplementary Figure S1**).

Clinical data collected included gender, age, TNM stage, adjuvant therapy after surgery, and survival outcomes. The computed tomography (CT) evaluations were based on the Response Evaluation Criteria in Solid Tumors (RECIST 1.1) and assessed by experienced radiologists.

### 2.2 Histopathologic assessment

**Figure 1:**
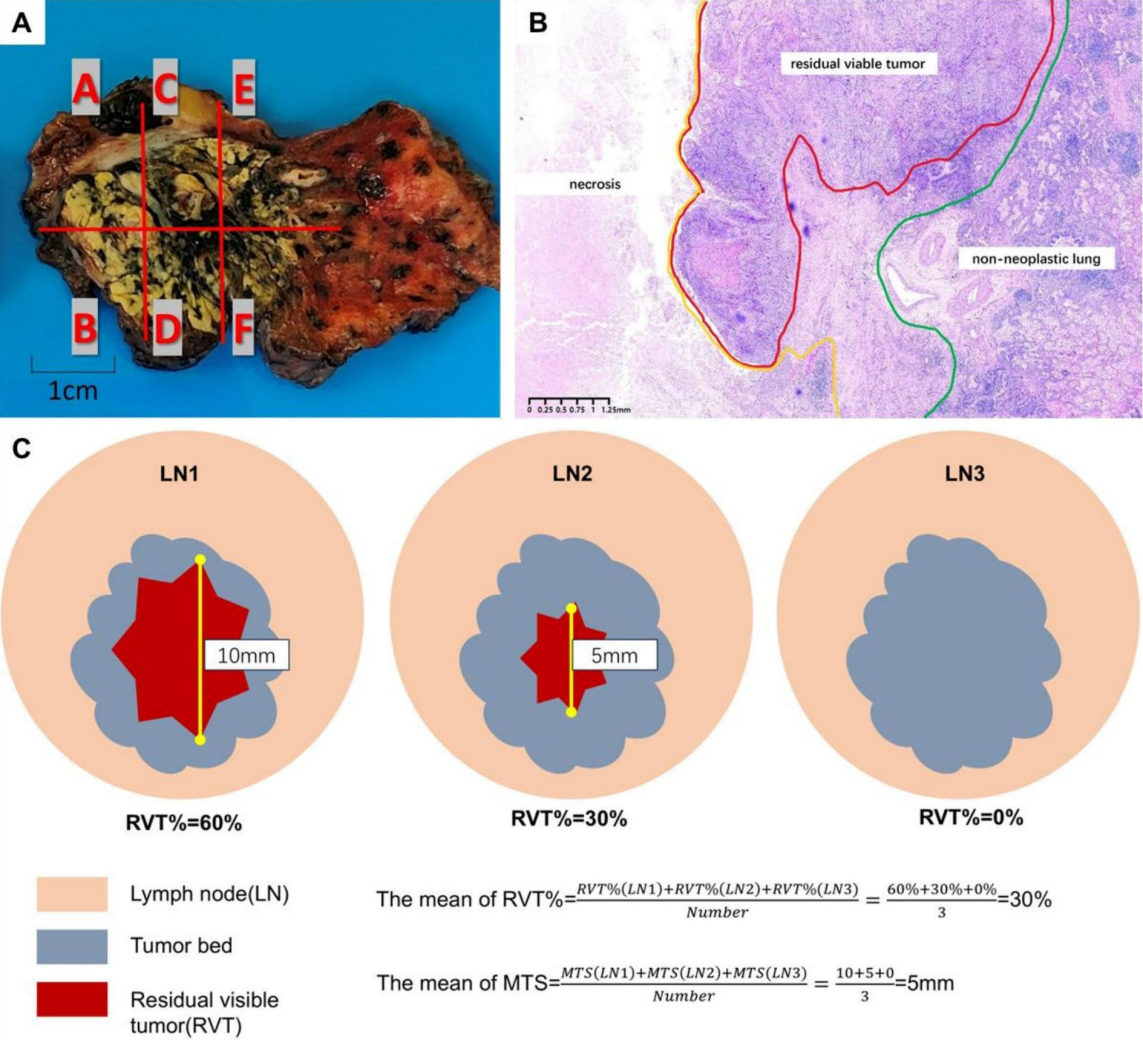
Pathologic assessment. **(A)** Gross appearance and mapping. This tumor shows a variegated cut surface with yellow necrotic areas and demonstrates the anatomical relationship of the tumor to the proximal bronchus and the overlying pleura. **(B)** Representative H&E slides for estimation of the residual tumor. Green: demarcation between the tumor bed and adjacent non-neoplastic lung, Red: demarcation of residual viable tumor, Yellow: demarcation of necrosis. **(C)** Schematic diagram of the histologic evaluation of lymph node specimens from patients with NSCLC after neoadjuvant chemoimmunotherapy. There may be several metastatic lymph nodes in one patient. Firstly, we calculated RVT% and MTS in each lymph node. Then, the mean of RVT% and MTS was the average of all the lymph nodes with metastatic tumor or complete remission. NSCLC, non-small cell lung cancer; RVT%, the percentage of residual viable tumor; MTS, metastatic tumor size.

The histologic subtype classification of NSCLC followed the 5th edition of the WHO Classification of Tumors of the Lung, Pleura, Thymus, and Heart (11). Gross examination and pathologic assessment adhered to the recommendations provided by the International Association for the Study of Lung Cancer (IASLC) (6). When the tumor bed’s maximum diameter did not exceed 3 cm, the entire tumor bed was sampled (**Figure 1A**). For tumor beds larger than 3 cm, a minimum of one block per centimeter of the tumor bed’s diameter was sampled. The median number of blocks of primary tumor reviewed per case was 9. All resected lymph nodes were sampled. If the lymph node’s maximum diameter exceeded 2 cm, it was bisected, and all sections were sampled. The median number of lymph node blocks reviewed per case was 11.

According to Cottrell’s study (11), treatment-related changes in the primary tumor and lymph node metastases include immune activation (tumor-infiltrating lymphocytes, tertiary lymphoid structures, plasma cells, granuloma), tumor cell death (foamy macrophages, cholesterol clefts, necrosis), and tissue repair (fibrosis, neovascularization) (**Supplementary Figure S2**). Following the IASLC recommendations (6), two experienced pathologists assessed the percentage of viable tumor cells, necrosis, and fibrosis in the tumor bed in 10% increments, unless the amount was less than 5% (**Figure 1B**). Pathological complete response (PCR) was defined as 0% residual visible tumor (RVT) in the primary tumor and dissected lymph nodes, while major pathologic response (MPR) was defined as ≤10% RVT in the primary tumor (6). The estimation of RVT percentage and metastatic tumor size (MTS) in lymph nodes followed our previous study (12). RVT% was calculated as the ratio of residual tumor area to tumor bed area in lymph nodes, mirroring the primary tumor method (6). MTS was measured directly with a ruler or a micrometer eyepiece reticle for smaller tumors under a microscope. For larger tumors, the maximum diameter was marked and measured with a ruler (12). In cases of dispersed tumors, size was the total of all metastatic foci’s cross-sectional areas. Each patient’s LNM was assessed for average RVT% and MTS (**Figure 1C**). The “negative” status indicates the absence of residual cancer and treatment response in the lymph nodes. In the derivation cohort, pathologic evaluations were conducted by S.W. and L.X.Y., while in the external validation cohort, Q.L.L. and X.Y. performed the assessments.

### 2.3 ypT staging and ypN staging assessment

Tumor staging was performed according to the 8th edition of the TNM classification system by the American Joint Committee on Cancer (AJCC) (13). As per the IASLC recommendations, the ypT stage is determined based on the size of the residual visible tumor, rather than the dimensions of the tumor bed (6). Additional factors influencing the ypT category, such as visceral pleura invasion and chest wall invasion, were also evaluated. The classification of visceral pleural invasion followed the criteria outlined in Shimizu et al.’s study (14).

The definitions for ypN staging were as follows:

ypN0: No residual tumor in the resected lymph nodes.
ypN1: Metastases in ipsilateral intrapulmonary, interlobar, or hilar lymph nodes.
ypN2: Metastases in ipsilateral mediastinal and/or subcarinal lymph nodes.
ypN3: Metastases in contralateral mediastinal, contralateral hilar, or supraclavicular lymph nodes.

### 2.4 Statistical analysis

The differences between categorical variables were assessed using the Chi-square test or Fisher’s exact test where appropriate. For correlations between ordinal or numerical variables, Spearman’s rank correlation was applied. Event-free survival (EFS) was defined as the time from the start of treatment to the first occurrence of either local or distant tumor recurrence or death from any cause. Kaplan–Meier plots were used to visualize survival data, with p-values calculated using the log-rank test.

Cox regression analysis was used for both univariate and multivariate analyses. The hazard ratio (HR) and 95% confidence intervals (CIs) were estimated using the Cox univariate model. Factors with p-values less than 0.10 in the univariate analysis were included in the multivariate analysis.

A nomogram model (15) was generated using the R package ‘rms’ to predict recurrence. Variables that were significant at P < 0.05 in the multivariate analysis were incorporated into the nomogram. A higher area under the curve (AUC) indicated better concordance between the model’s predictions and actual outcomes.

All statistical analyses were performed using IBM SPSS Statistics 20.0 (IBM Corp., Armonk, NY, USA) and R version 4.3.1 (The R Project for Statistical Computing, Vienna, Austria). P-values less than 0.05 were considered statistically significant.

## 3 Results

### 3.1 Clinical features

A total of 299 patients with NSCLC who underwent NCI were included in this study. Of these, 208 patients were part of the derivation cohort, while 91 patients were included in the external validation cohort. Part of the study population was included in our previous work (12). The clinical and histological characteristics of both cohorts are summarized in **Table 1**.

**Table 1:** Clinical and histologic characteristics of patients.

Characteristic	Derivation cohort (n=208)	External validation cohort (n=91)	*P *value
Gender			0.277
Male	186 (89.4%)	85 (93.4%)	
Female	22 (10.6%)	6 (6.6%)	
Age			0.094
<60	90 (43.3%)	30 (33.0%)	
≥60	118 (56.7%)	61 (67.0%)	
Smoking			
Yes	178 (85.6%)	60 (65.9%)	<0.001*
No	30 (14.4%)	31 (34.1%)	
Adjuvant therapy			0.170
Yes	74 (35.6%)	40 (44.0%)	
No	134 (64.4%)	51 (56.0%)	
Histological subtypes			0.538
Squamous cell carcinoma	157 (75.5%)	63 (69.2%)	
Adenocarcinoma	41(19.7%)	22 (24.2%)	
Adenosquamous carcinoma	5 (2.4%)	3 (3.3%)	
Combined large cell neuroendocrine carcinoma	2 (1.0%)	0 (0%)	
Large cell neuroendocrine carcinoma	1 (0.5%)	2 (2.2%)	
Sarcomatoid carcinoma	1 (0.5%)	1 (1.1%)	
NSCLC, NOS	1 (0.5%)	0 (0%)	
Pleural invasion			0.660
Yes	9 (4.3%)	5 (5.5%)	
No	199 (95.7%)	86 (94.5%)	
ypT staging			0.508
ypT0	97 (46.6%)	49 (53.8%)	
ypT1	81 (38.9%)	27 (29.7%)	
ypT2	17 (8.2%)	8 (8.8%)	
ypT3	11 (5.3%)	5 (5.5%)	
ypT4	2 (1.0%)	2 (2.2%)	
ypN staging			0.487
ypN0	158 (76.0%)	67 (73.6%)	
ypN1	30 (14.4%)	11 (12.1%)	
ypN2/3	20 (9.6%)	13 (14.3%)	
MPR			0.025*
Yes	130 (62.5%)	69 (75.8%)	
No	78 (37.5%)	22 (24.2%)	
Pathology response in lymph node			0.420
Negative	124 (59.6%)	49 (53.8%)	
MTS≤4.5mm	73 (35.1%)	34 (37.4%)	
MTS>4.5mm	11 (5.3%)	8 (8.8%)	
Survival			0.609
No recurrence	172 (82.7%)	73 (80.2%)	
Recurrence or death	36 (17.3%)	18 (19.8%)	

*p<0.05

MPR, major pathologic response; yp, pathological staging after neoadjuvant therapy.

The derivation cohort had a significantly higher proportion of smokers compared to the external validation cohort (p < 0.001). Although more patients in the external validation cohort achieved MPR (*p* = 0.025), there were no statistically significant differences between the two cohorts in terms of clinical outcomes (*p* = 0.609).

Other clinical and histological variables, such as gender, age, adjuvant therapy following surgery, histological subtypes, presence of pleural invasion, ypT and ypN staging, and pathological response in lymph nodes, did not show significant differences between the two groups.

### 3.2 Relationship between ypT staging and the depth of pathologic response

**Figure 2:**
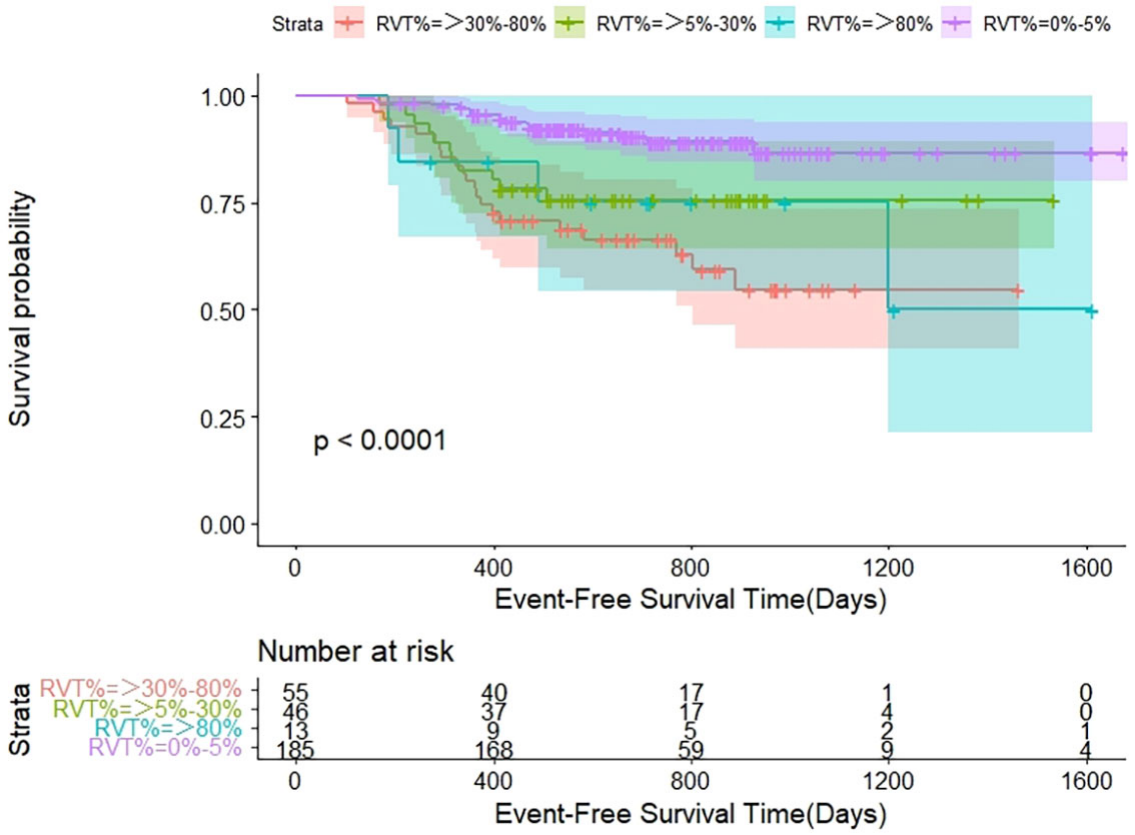
The relationship between patient survival and the depth of pathological response. The patients’ survival was significantly associated with the depth of pathologic response, which were subgrouped by the RVT% of primary tumor. RVT%, the percentage of residual viable tumor.

According to the CheckMate 816 trial, the depth of pathologic response was significantly associated with event-free survival (EFS) (9). Patients were subgrouped based on the residual visible tumor percentage (RVT%) in the primary tumor: 0% to 5%, >5% to 30%, >30% to 80%, and >80%. Our results similarly showed a significant association between these subgroups and EFS (**Figure 2**).

The relationship between ypT staging and the depth of pathologic response is presented in **Table 2**. There was a strong correlation between ypT staging and pathologic response (Spearman’s test, r = 0.81; 95% CI, 0.76-0.84; *p* < 0.0001), although an absolute association could not be conclusively established. Among patients with ypT3/4 staging, 35.0% (7/20) had an RVT% >80%, and 50.0% (10/20) had an RVT% between >30% and 80%. Three patients were classified as ypT3 due to chest wall invasion, while one patient was classified as ypT4 due to the presence of separate tumor nodules in a different ipsilateral lobe.

**Table 2:** Relationship between ypT staging and the depth of pathologic response.

RVT% in primary tumor	Derivation cohort (n=208)				External validation cohort (n=91)			
	ypT0	ypT1	ypT2	ypT3/4	ypT0	ypT1	ypT2	ypT3/4
0% to 5%	97	26	0	0	49	12	1	0
>5% to 30%	0	29	1	0	0	10	3^$^	3^#^
>30% to 80%	0	24	12*	6^#^	0	5	4^%^	4^&^
>80%	0	2	4	7	0	0	0	0

* Four cases are classified as ypT2 based on visceral pleura invasion, rather than the size of residual visible tumor.

^$^ One of these cases is classified as ypT2 based on direct tumor invasion into an adjacent ipsilateral lobe, and another is classified as ypT2 based on visceral pleura invasion.

^#^ This case is classified as ypT3 based on chest wall invasion, rather than the size of residual visible tumor.

^%^ Two of these cases is classified as ypT2 based on visceral pleura invasion, rather than the size of residual visible tumor.

^&^ One case is classified as ypT3 based on chest wall invasion, and another is classified as ypT4 due to separate tumor nodules in a different ipsilateral lobe.

### 3.3 Prognostic factors related to EFS in the derivation cohort

In the univariate analysis, event-free survival (EFS) showed no statistically significant association with gender (male vs. female, *p* = 0.49), age (≤60 vs. >60, *p* = 0.89), histological subtypes (non-SCC vs. SCC, *p* = 0.31), smoking status (yes vs. no, *p* = 0.52), or adjuvant therapy (yes vs. no, *p* = 0.50). However, MPR (yes vs. no, *p* = 0.00018), pathological response in lymph nodes (negative vs. MTS ≤4.5 mm vs. MTS >4.5 mm, *p* < 0.0001), ypT staging (ypT0/2 vs. ypT3/4, *p* < 0.0001), and ypN staging (ypN0 vs. ypN1 vs. ypN2/3, *p* = 0.0047) were significantly associated with EFS.

In the multivariate analysis, the following were identified as independent adverse prognostic factors for EFS:

MPR (no vs. yes; HR = 2.860; 95% CI, 1.245–6.567; *p* = 0.013)
ypT staging (ypT3/4 vs. ypT0/2; HR = 3.987; 95% CI, 1.496–10.629; *p* = 0.006)

Pathological response in lymph nodes:

MTS ≤4.5 mm vs. Negative; HR = 4.059; 95% CI, 1.558–10.571; *p* = 0.004
MTS >4.5 mm vs. Negative; HR = 6.871; 95% CI, 1.713–27.564; *p* = 0.007
These findings are summarized in **Table 3**.

**Table 3:** Multivariate analysis for event-free survival in the derivation cohort.

Risk factor	HR	95% confidence interval	*P*
ypN staging			0.173
ypN0	reference	reference	
ypN1	0.366	0.112-1.198	0.097
ypN2/3	0.821	0.262-2.572	0.735
MPR			0.013*
Yes	Reference	Reference	
No	2.860	1.245-6.567	
ypT staging			0.006*
ypT0-2	Reference	Reference	
ypT3-4	3.987	1.496-10.629	
Pathology response in lymph node			0.009*
Negative	Reference	Reference	
MTS≤4.5mm	4.059	1.558-10.571	0.004*
MTS>4.5mm	6.871	1.713-27.564	0.007*


*p<0.05.

MPR, major pathologic response; yp, pathological staging after neoadjuvant therapy.

### 3.4 Construction and evaluation of nomogram

**Figure 3:**
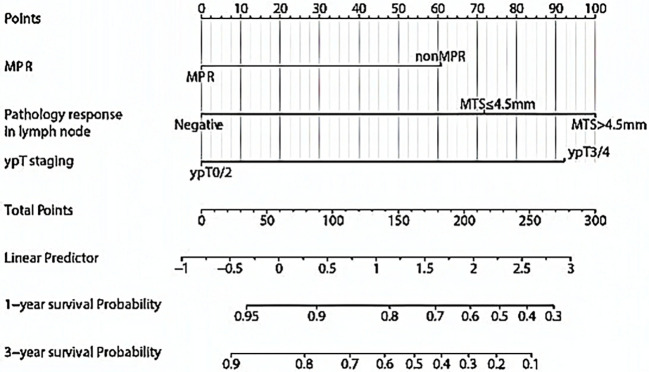
Nomogram for estimating survival in patients with NSCLC undergoing neoadjuvant chemoimmunotherapy. The nomogram integrates three critical variables: ypT staging, MPR, and pathological response in lymph nodes. Each variable is aligned with a scoring axis, where the status of each factor corresponds to a specific score. The sum of the scores from all variables yields a total score, which is subsequently mapped onto a prediction line below. NSCLC, non-small cell lung cancer; MPR, major pathologic response; MTS, metastatic tumor size.

Based on the independent predictors identified through multivariate analysis, we developed a predictive nomogram model for event-free survival (EFS) in NSCLC patients following NCI (**Figure 3**). The nomogram incorporates three key variables: ypT staging, MPR, and pathological response in lymph nodes. Each variable is aligned with a score axis, where the state of each factor corresponds to a specific score. The cumulative score from all the variables provides a total score, which is then mapped onto a prediction line below. This line offers the corresponding predicted survival probability.

### 3.5 Discrimination of the nomogram

**Figure 4:**
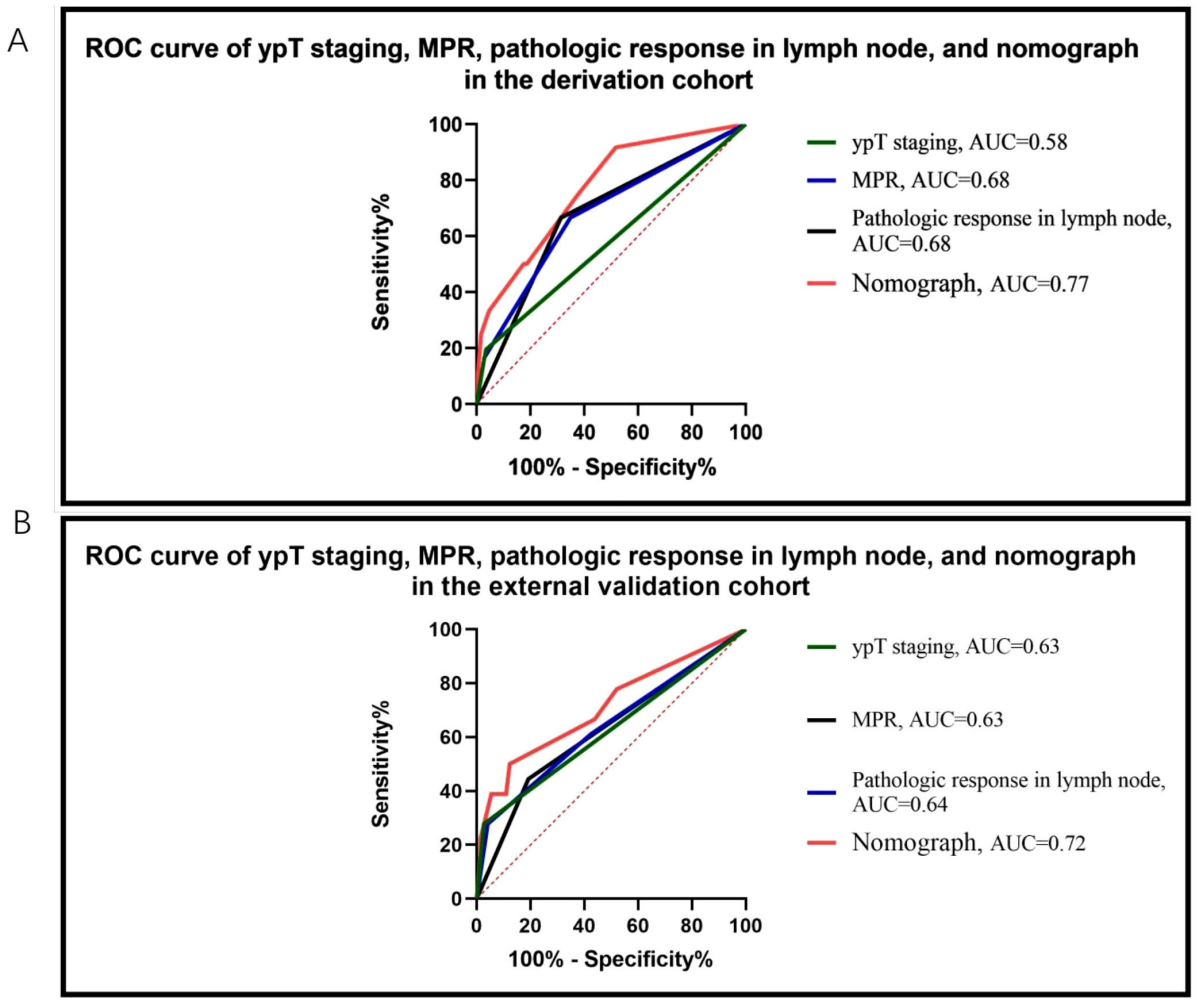
ROC curve of ypT staging, MPR, pathologic response in lymph node, and nomograph in the derivation cohort **(A)** and external validation cohort **(B)**. The nomograph exhibited superior predictive performance for survival compared to individual predictors such as ypT staging, MPR, and lymph node pathology response, achieving the highest AUC values in both cohorts. ROC, receiver operating characteristic; MPR, major pathologic response; AUC, area under curve.

The nomogram achieved an area under the curve (AUC) of 0.77 (95% confidence interval [CI] = 0.69–0.85) in the derivation cohort (**Figure 4A**) and 0.72 (95% CI = 0.56–0.86) in the external validation cohort (**Figure 4B**). It demonstrated superior predictive performance for event-free survival (EFS) compared to individual predictors such as ypT staging, MPR, and lymph node pathology response, yielding the highest AUC values in both cohorts.

### 3.6 Risk Stratification System

**Figure 5:**
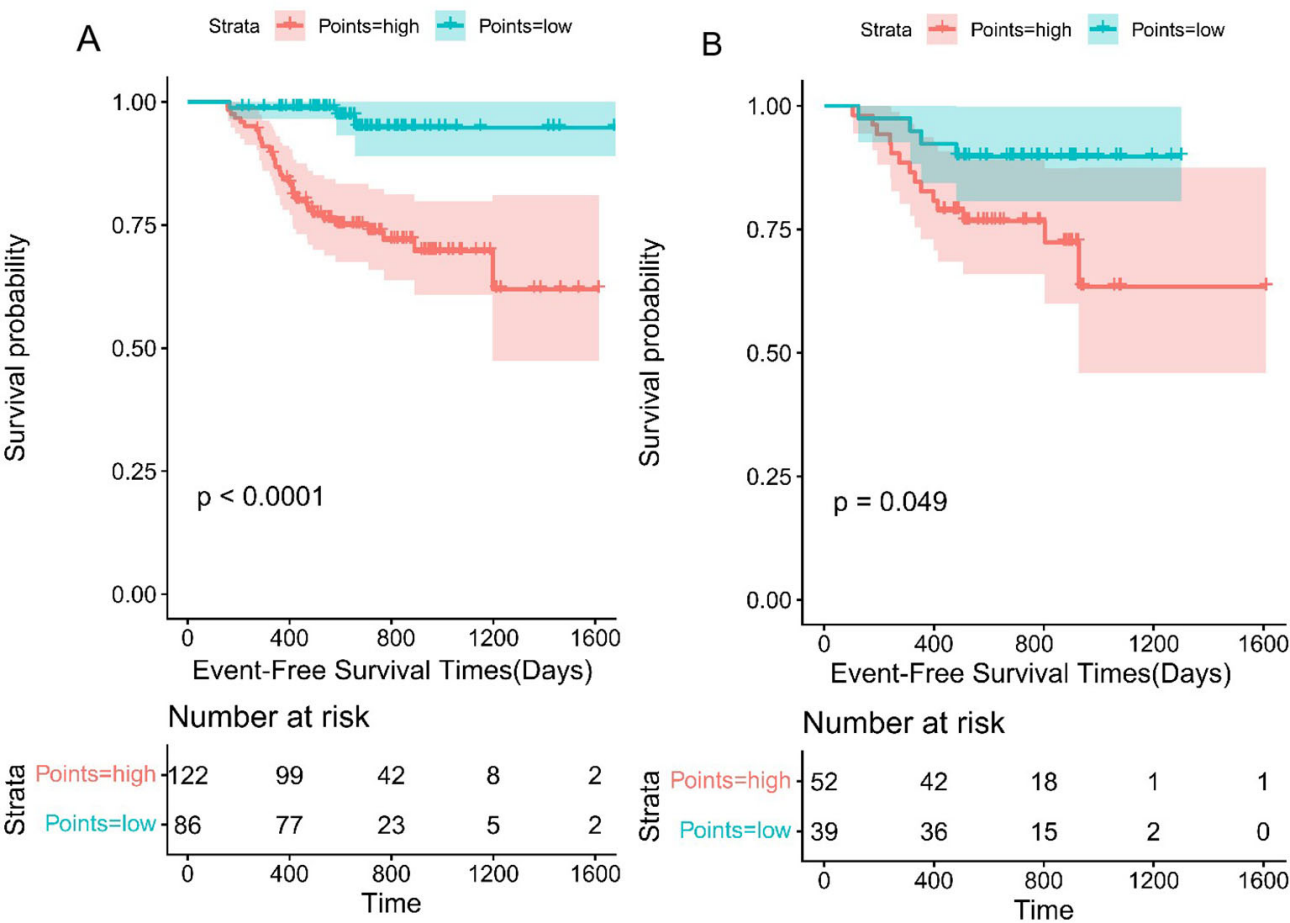
Kaplan-Meier curve to test the stratification system in the training cohort **(A)** and validation set **(B)**.

The above analyses demonstrated the robust predictive performance of the survival nomogram. Prediction scores were computed based on the three variables incorporated in the nomogram. An optimal cutoff value was established to stratify patients in the training cohort into low-risk (risk score ≤ 0) and high-risk (risk score > 0) groups. Kaplan-Meier survival analysis revealed that the EFS of low-risk patients (n = 86) was significantly better than the high-risk patients (n = 122) (P <0.0001) (**Figure 5A**). Furthermore, Kaplan-Meier analysis conducted within the validation cohort confirmed the utility of this risk stratification system, showing that EFS was significantly higher in low-risk patients (n = 39) relative to high-risk patients (n = 52) (P < 0.05) (**Figure 5B**).

The application of the nomogram was as follows:

1. Fundamental steps for interpreting the nomogram:

Variable alignment: Identify the patient’s actual clinical data points on the corresponding variable axes of the nomogram (e.g., the mean MTS of lymph nodes, MPR, ypT), and record the associated values on the “score axis” for each variable.

Total score computation: Aggregate the scores from all variables to derive the patient’s overall “total risk score”, then locate this score on the nomogram’s “total score axis”.

Risk estimation: Using the total risk score, determine the predicted probability on the “outcome axis” (such as 1-year or 3-year EFS). This probability reflects the average risk level among a cohort with comparable clinical characteristics.

For example, a patient classified as ypT2 (0 points) with negative MTS (0 points) who achieved MPR (0 points) accumulated a total predictive score of 0 + 0 + 0 = 0 points. This aggregate score corresponded to an estimated 3-year survival probability of approximately 83% (**Figure 3**).

2. Critical considerations for application in adjuvant therapy decision-making:

Risk stratification to guide therapeutic choices: Patients classified as “low risk” (total score ≤ 0) are generally recommended for observation and routine follow-up to prevent overtreatment; conversely, those identified as “high risk” (total score > 0) may benefit from more aggressive adjuvant interventions, such as combined chemotherapy and immunotherapy.

3. Integration with clinical context:

Nomogram predictions should be interpreted alongside the patient’s comorbid conditions (e.g., cardiopulmonary, hepatic, and renal function), treatment tolerance, and personal preferences. For instance, a high-risk patient with significant cardiopulmonary impairment who is unable to tolerate combination therapy might, following comprehensive discussion, be managed with monotherapy and increased surveillance frequency.

## 4 Discussion

Pathologic response and the ypTNM system (pathological staging after neoadjuvant therapy) are regarded as crucial postoperative prognostic factors. However, further research is needed to evaluate the prognostic value of ypT staging, ypN staging, MPR, and the pathological assessment of lymph nodes in the context of NCI.

Previous studies have demonstrated that combining multiple prognostic factors can effectively stratify patients with resected NSCLC without adjuvant nor neoadjuvant therapy into different risk categories (16, 17). For instance, Liang et al. developed a nomogram incorporating factors such as gender, age, number of resected lymph nodes, histologic type, T staging, and N staging, which outperformed traditional TNM staging in predicting survival (16). Similarly, Pilotto et al. (17) devised a combined stratification model for lung SCC, incorporating age, tumor grading, T staging, and lymph node status.

This model, validated through multicenter external validation, demonstrated prognostic value that could be applied to both adjuvant and neoadjuvant chemotherapy (18). Zens et al. also established a prognostic score for NSCLC after neoadjuvant chemotherapy, combining MPR, ypN staging, and ypT staging, which accurately predicted overall survival (OS) and disease-free survival (DFS) (19). However, incorporating tumor grading into prognostic models for NSCLC after neoadjuvant therapy remains challenging, because tumor morphology is too altered to be graded accurately after neoadjuvant therapy (11). To date, there is no literature on the application of prognostic models for NCI. In this study, we established a prognostic nomogram combining MPR, ypT staging and pathological assessment of lymph node for NCI predicting EFS more accurately than the TNM staging system or MPR alone. Our prognostic model offers a simple and accessible tool for clinical practice.

The prognostic value of ypT staging in the neoadjuvant setting remains uncertain. To date, no universally accepted protocol for evaluating ypT staging exists, leading to potential variations in results depending on the method used to estimate residual tumor volume (20). Notably, according to the International Association for the Study of Lung Cancer (IASLC) recommendations, ypT staging depends on the measurement of the size of the residual visible tumor (RVT) (6), indicating a close relationship between ypT staging and the depth of pathologic response. In addition to residual tumor size, factors such as visceral pleura invasion and chest wall invasion also influence ypT staging (13). Both pathologic response and ypT staging focus mainly on the primary tumor after surgery, raising the question of whether pathologic response alone can replace ypT staging in predicting survival outcomes. In this study, we evaluated ypT staging according to the IASLC recommendations (6) and the 8th edition AJCC TNM classification (13). While pathologic response outperformed ypT staging in predicting EFS (as shown by a higher AUC in the derivation cohort), it cannot fully replace ypT staging, particularly for advanced cases. Several factors, such as chest wall invasion or separate tumor nodules in different ipsilateral lobes, influence ypT staging beyond just residual tumor size (**Table 2**).

Furthermore, previous studies have demonstrated a significant association between the depth of pathologic response and survival (9, 21). In the CheckMate 816 trial, patients with an RVT% greater than 80% had the lowest 2-year EFS rates (9). Our results were consistent with these findings (**Figure 2**). In our study, 38.9% of patients with ypT3/4 had an RVT% greater than 80%, and 55.6% had an RVT% between 30% and 80%. These findings underscore the strong association between ypT staging and EFS, with advanced ypT staging serving as an independent adverse prognostic factor.

In contrast, ypN staging has been established as an independent prognostic factor in NSCLC treated with neoadjuvant chemotherapy (22, 23) and chemoradiotherapy (24). Both MPR and ypN staging significantly impact post-surgical prognosis. Studies have shown that only patients with MPR and ypN0 (no lymph node involvement) achieve prolonged disease-free survival (DFS) compared to those with MPR and ypN1-2 (lymph node involvement) in neoadjuvant chemotherapy (25, 26). However, findings from NCI may differ. According to the CheckMate 816 trial, the achievement of MPR in the primary tumor is associated with improved EFS regardless of lymph node involvement (9).

In terms of lymph node pathology, previous studies have suggested that pathologic response in lymph nodes may be a crucial prognostic factor, potentially more valuable than ypN staging alone (27–29). For example, Liu et al. identified RVT ≤ 8% in lymph nodes as an independent predictor of improved DFS after neoadjuvant chemotherapy (28). Pataer et al. reported that RVT ≤ 70% in lymph nodes was a favorable prognostic factor for OS (27). Additionally, Deng et al. found that RVT ≤ 10% in lymph nodes predicted better DFS following NCI (29). Furthermore, our previous study suggested that the mean metastatic tumor size (MTS) is a more effective parameter for pathological evaluation of lymph nodes than ypN stage, with a threshold of 4.5 mm being closely associated with EFS (12). This suggests that ypN staging may need to be modified based on pathologic response in lymph nodes.

In this study, we evaluated pathologic response, ypT staging, ypN staging, and the mean MTS of lymph nodes, incorporating them into our Cox regression analyses. Our findings confirmed that pathologic response, ypT staging, and mean MTS were independent prognostic factors for these patients, acting as strong predictors of EFS. The nomogram model we developed further confirmed the predictive performance of these histopathological features in both the derivation and external validation cohorts. Based on these results, we propose that MPR, ypT staging, and mean MTS of lymph nodes hold significant prognostic value.

However, there are several limitations to this retrospective study. The survival endpoint used was EFS. While this endpoint provides valuable insights, a longer follow-up period is necessary to confirm and validate our results. A recent study by Donington et al. highlighted the importance of EFS as a strong predictor of OS in NSCLC patients treated with neoadjuvant therapy (30). Moreover, since NCI is a relatively novel treatment approach, its long-term effectiveness and associated prognostic factors are still being explored. Another limitation concerns the potential variability in pathological assessments performed across multiple institutions and by different clinicians. Consequently, it is imperative to strive for the highest possible standardization of these evaluations. Furthermore, additional research is warranted to investigate the influence of postoperative treatment strategies on patient prognosis. To the best of our knowledge, this study represents the first attempt to develop a combined prognostic model for NSCLC patients treated with NCI. Further prospective studies with longer follow-up periods are needed to validate our findings and refine the model.

In conclusion, this study assessed the prognostic significance of ypT staging, ypN staging, MPR, and mean MTS of lymph nodes in NSCLC patients following NCI. Our findings suggest that combining ypT staging, MPR, and mean MTS offers valuable insights for predicting survival outcomes in this patient population.

## Data Availability

The original contributions presented in the study are included in the article/**Supplementary Material**. Further inquiries can be directed to the corresponding authors.

## Supplementary material

Supplementary figure S1 Flow diagram of patients included in the study.

Supplementary figure S2 The treatment related changes. **(A)** tumor infiltrating lymphocytes and proliferative fibrosis, **(B)** tertiary lymphoid structure, **(C)** cholesterol cleft, **(D)** foamy macrophage, **(E)** necrosis, **(F)** neovascularization (labeled by black arrow).
