# Supplementary figures and images for: Development and external validation of a prognostic nomogram for event-free survival in resectable non-small cell lung cancer after neoadjuvant chemoimmunotherapy
Source: Front Oncol. 2025 Dec 16;15:1682497. doi: 10.3389/fonc.2025.1682497 (PMC12747836; doi:10.3389/fonc.2025.1682497)

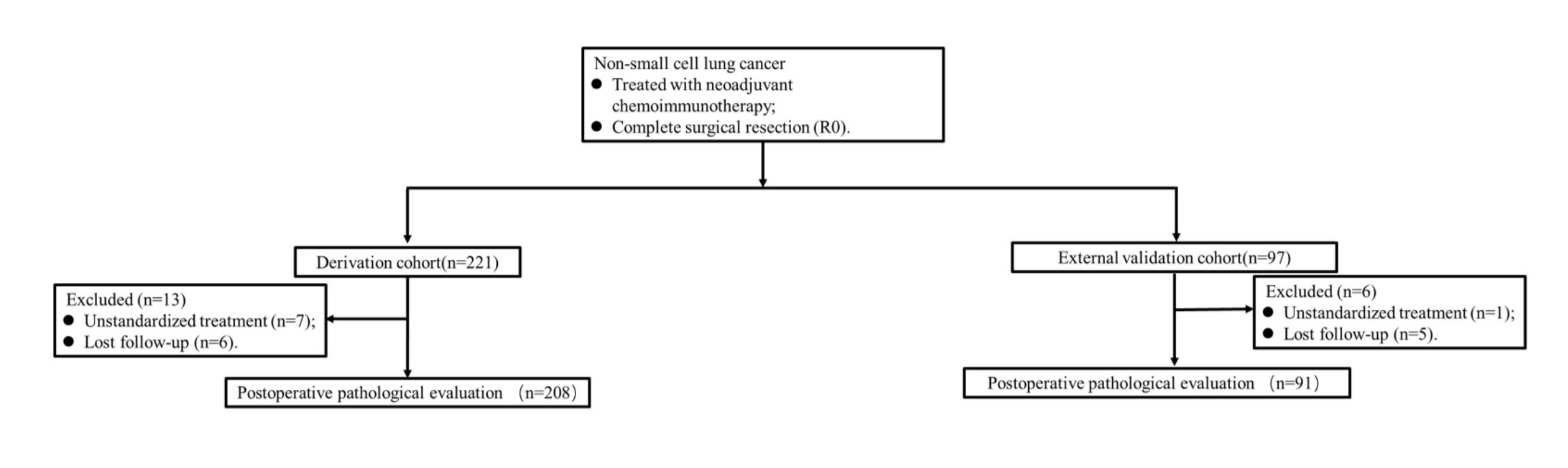

Supplement: Supplementary figure S1 — Flow diagram of patients included in the study. [file Image1.jpeg]

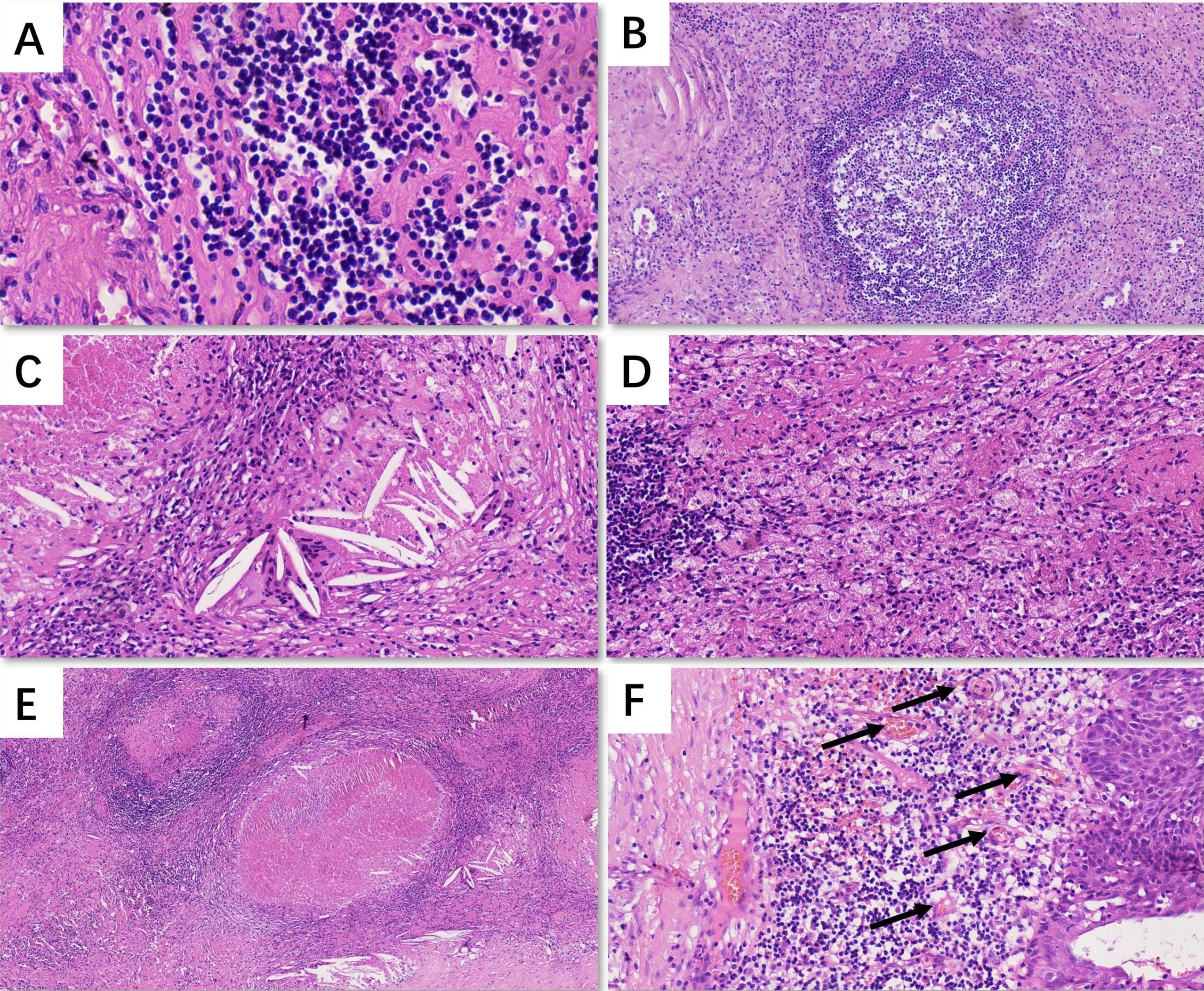

Supplement: Supplementary figure S2 — The treatment related changes. (A) tumor infiltrating lymphocytes and proliferative fibrosis, (B) tertiary lymphoid structure, (C) cholesterol cleft, (D) foamy macrophage, (E) necrosis, (F) neovascularization (labeled by black arrow). [file Image2.jpeg]
